# High throughput sequencing provides exact genomic locations of inducible prophages and accurate phage-to-host ratios in gut microbial strains

**DOI:** 10.1186/s40168-021-01033-w

**Published:** 2021-03-29

**Authors:** Mirjam Zünd, Hans-Joachim Ruscheweyh, Christopher M. Field, Natalie Meyer, Miguelangel Cuenca, Daniel Hoces, Wolf-Dietrich Hardt, Shinichi Sunagawa

**Affiliations:** 1 Department of Biology, Institute of Microbiology and Swiss Institute of Bioinformatics, ETH Zürich, Zürich, Switzerland; 2 Department of Health Sciences and Technology, Institute of Food, Nutrition and Health, ETH Zürich, Zürich, Switzerland

**Keywords:** Prophage localisation, Prophage induction, Phage activity, Phage replication, High-throughput sequencing, Phage-to-host ratio, Gut microbiota

## Abstract

**Background:**

Temperate phages influence the density, diversity and function of bacterial populations. Historically, they have been described as carriers of toxins. More recently, they have also been recognised as direct modulators of the gut microbiome, and indirectly of host health and disease. Despite recent advances in studying prophages using non-targeted sequencing approaches, methodological challenges in identifying inducible prophages in bacterial genomes and quantifying their activity have limited our understanding of prophage-host interactions.

**Results:**

We present methods for using high-throughput sequencing data to locate inducible prophages, including those previously undiscovered, to quantify prophage activity and to investigate their replication. We first used the well-established *Salmonella enterica* serovar Typhimurium/p22 system to validate our methods for (i) quantifying phage-to-host ratios and (ii) accurately locating inducible prophages in the reference genome based on phage-to-host ratio differences and read alignment alterations between induced and non-induced prophages. Investigating prophages in bacterial strains from a murine gut model microbiota known as Oligo-MM^12^ or sDMDMm2, we located five novel inducible prophages in three strains, quantified their activity and showed signatures of lateral transduction potential for two of them. Furthermore, we show that the methods were also applicable to metagenomes of induced faecal samples from Oligo-MM^12^ mice, including for strains with a relative abundance below 1%, illustrating its potential for the discovery of inducible prophages also in more complex metagenomes. Finally, we show that predictions of prophage locations in reference genomes of the strains we studied were variable and inconsistent for four bioinformatic tools we tested, which highlights the importance of their experimental validation.

**Conclusions:**

This study demonstrates that the integration of experimental induction and bioinformatic analysis presented here is a powerful approach to accurately locate inducible prophages using high-throughput sequencing data and to quantify their activity. The ability to generate such quantitative information will be critical in helping us to gain better insights into the factors that determine phage activity and how prophage-bacteria interactions influence our microbiome and impact human health.

Video abstract.

**Supplementary Information:**

The online version contains supplementary material available at 10.1186/s40168-021-01033-w.

## Background

The gut microbiome of humans and other mammals is composed of a diverse range of eukaryotic microorganisms, archaea, bacteria and viruses [1]. Compared to the different types of microorganisms identified in the gut, viruses that infect bacteria (phages) are suggested to be numerically dominant, highly diverse, individual-specific and stable over time [2–5]. Of the gut phages that have been identified to date, many display a ‘temperate’, lysogenic life cycle in which they replicate as either genome-integrated or extrachromosomal prophages within host bacterial cells [6]. Upon induction, temperate phages can enter the lytic cycle, during which they replicate within the host cell and produce new infective particles (virions) that are released upon lysis of the host cell [7].

The widespread manifestation of lysogeny amongst phages found in the gut microbiome suggests an important role for prophages in controlling the density, diversity and function of gut bacterial populations [8–10]. Prophages can be induced to enter the lytic cycle either spontaneously [11], or by stress stimuli [12–15]. The subsequent production and release of virions is usually lethal to bacterial host cells, and may also impact non-susceptible species within a gut bacterial community through cascading effects that owe to the disturbance caused to bacterial interaction networks [16]. The activity and abundance of phages may thus cause substantial alterations to gut microbial community compositions [17] and in turn, impact host-microbe interactions. Indeed, phages are increasingly recognised to have an essential role in the health and disease of mammals. Alteration in the abundance and composition of viral populations have been detected, for example, in the context of gastrointestinal disorders [18–20]; after dietary perturbation in humans [21] and mice [22, 23], as well as in response to antibiotic administration in pigs [14].

Whilst the importance of prophages as mobile genetic elements [24] and modulators of the gut microbiome [8] is now evident, methodological challenges in identifying prophages in bacterial genomes and in assessing their inducibility still limit our understanding of prophage-host interactions. For example, studying prophages can be laborious when relying on targeted cultivation- and imaging-based approaches [25, 26]. As an alternative, recent advances have enabled researchers to identify and characterise phages by analysing high-throughput sequencing (HTS) data, including in uncultured gut microbial community (i.e. faecal) samples [2, 27]. One approach is to enrich virions from cultures or communities of host bacteria, extract the DNA of viral genomes and subject it to shotgun metagenomic sequencing. This approach makes it possible to reconstruct entire viral genomes from shotgun sequence data, has led to increased expansion of viral genome sequences in public databases [28] and provided an improved ability to identify active prophage producing virions [29]. However, the choice of enrichment methods was shown to influence the number of recovered virus types [30] and the low yield of DNA isolated from virions usually necessitates an additional amplification step, which is prone to generating uneven or chimeric representations of genomic fragments [31, 32]. Together, the pre-treatment steps before virion sequencing thus commonly lead to biases in the relative abundance estimation of viruses.

To overcome the limitation of low DNA yields from enriched virions, an alternative approach is to extract DNA from whole cultures or community samples without an enrichment step and shotgun sequence the entire community. Using this approach, the activity of prophages in the entire culture can be quantified by assessing the relative copy number of phage genomes per host genome—known as the phage to host (PtoH) ratio. For example, using HTS data from human gut microbiome samples, Waller et al. (2014) sought to quantify PtoH ratios by aligning sequencing reads to prophage regions in sequenced reference genomes [33]. This approach relies on the use of bioinformatics tools, such as VirSorter [34], VIBRANT [35], PHASTER [36] and Prophage Hunter [37], which predict prophage regions in fragmented or complete microbial genomes by using information from virus databases and testing for the presence of viral hallmark genes [38]. However, these methods rely on similarity searches within existing databases or machine learning using known phage sequences for training. This may bias the prediction of novel prophages in favour of those that are most similar to previously reported prophages. Furthermore, to our knowledge, no attempts have been made to assess the accuracy of different tools in predicting the genomic coordinates (i.e. locations) of prophages and to experimentally validate whether HTS-derived PtoH ratios are in congruence with established quantitative methods, such as quantitative PCR (qPCR).

Here, we describe the development and validation of methods (i) to test the inducibility, (ii) to determine the precise genomic location and (iii) to explore the replication mode of prophages, and (iv) to accurately quantify PtoH ratios. We subsequently applied these methods to identify, locate and characterise several inducible prophages in gut microbial model strains. We further show that this approach can also be used to analyse prophage activity in ex vivo-cultured gut microbial communities. Based on experimentally validated data, we finally discuss the variability amongst and limited accuracy of several prophage prediction tools.

## Results

### High throughput sequencing reflects accurate PtoH ratios in mitomycin C-induced *S*. Typhimurium cultures

As a model organism for prophage induction, we used a *Salmonella enterica* serovar Typhimurium LT2 strain (*S*. Tm LT2^*p22*^). In addition to its original prophages (FELS-1, FELS-2, Gifsy-1 and Gifsy-2), *S*. Tm LT2^*p22*^ carries the prophage p22 [39]. Treatment of *S*. Tm LT2^*p22*^ cultures with mitomycin C has previously been shown to result in DNA damage, leading to inactivation of prophage repressors [13, 40] and subsequent induction of p22 [13, 40]. The induction and replication mode of p22 involves the excision and subsequent circularisation of the phage genome by recombination of homologous sequences present at the attachment sites (*att*L and *att*R) of the prophage genome [41]. Upon excision of the phage genome, recombined attachment sites are generated in the phage (*att*P) and host (*att*B) genomes resulting in a circular phage genome and a naïve *S*. Tm LT2^*p22*^ strain not carrying the prophage p22 in its genome anymore. In the lytic cycle, concatemeric phage DNA is formed as a result of rolling circle amplification [42], which is packaged into capsids via a mechanism named headful packaging [43]. During this process, DNA is packaged into procapsids initiated from a *pac* site and proceeding unidirectionally until the carrying capacity of the first procapsid is reached before continuing with packaging DNA into the next procapsid starting wherever the previous one finished [43].

**Fig. 1:**
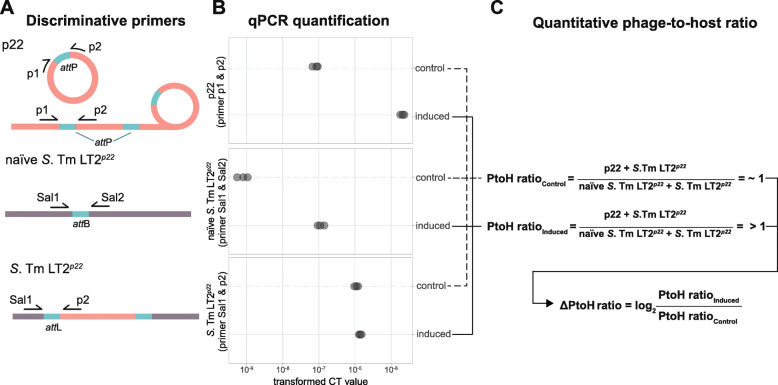
Establishment of quantitative reference values for the induction of prophage p22 in *S*. Tm LT2^*p22*^. **a** The schema illustrates the different primer combinations that were used to amplify genomic regions that are indicative of circularised and concatemeric prophage p22 genomes (top), naïve *S*. Tm LT2^*p22 *^genomes lacking the p22 prophage (middle) and lysogenic *S*. Tm LT2^*p22*^ genomes (bottom) owing to the primers targeting either bacterial (grey) or viral (red) DNA sequence present next to the attachment site sequences *att*P, *att*B and *att*L (turquoise), respectively. The three different primers pairs are shown as black arrows. Sal1 and Sal2 target bacterial and p1 and p2 viral DNA sequences. **b** Quantitative PCR readouts from non-induced (*n*=3, control) and mitomycin C-induced (*n*=3, induced) *S*. Tm LT2^*p22*^ cultures. The different primer combinations targeting *att*P, *att*B and *att*L allow for determining the quantity of naïve *S*. Tm LT2^*p22*^, *S*. Tm LT2^*p22*^ and induced p22 within a single culture. The transformed cycle threshold (CT) values are proportional to the amount of template DNA in each sample. The data suggest that upon induction, p22 and naïve *S*. Tm LT2^*p22*^ genome copy numbers increase as a consequence of p22 excision. A non-homogeneous induction of *S*. Tm LT2^*p22*^ cells in the treated culture with growth of non-induced *S*. Tm LT2^*p22*^ cells over the period of 24 h is most likely the explanation for unchanged CT values between control and induced *S*. Tm LT2^*p22*^ cultures. **c** PtoH ratios measure the activity of p22 by assessing the relative copy number of phage genomes per host genomes. To this end, we compared the amount of viral DNA from *S*. Tm LT2^*p22*^ (using primers Sal1 & p2) and induced p22 (using primers p1 & p2) genomes to the combined amount of bacterial DNA from *S*. Tm LT2^*p22*^ (using primers Sal1 & p2) and naïve *S*. Tm LT2^*p22*^ (using primers Sal1 & Sal2) genomes within control and induced cultures. PtoH differences were calculated as the mean log_2_-fold change (l_2_fc) between induced and control cultures (ΔPtoH ratio)

Using this system, we first established quantitative reference values for PtoH ratios by performing qPCR measurements using induced and non-induced (control) *S*. Tm LT2^*p22*^ cultures. We designed four qPCR primers (see the ‘Methods’ section), which, depending on their combination, are indicative of naïve *S*. Tm LT2^*p22*^ strains if flanking *att*B (both primers target bacterial DNA), integrated prophages if flanking *att*L (primers target bacterial and viral DNA) and circularised (or concatenated) p22 phage genomes if flanking *att*P (both primers target viral DNA) (Fig. 1a/b). In control cultures, we determined a PtoH ratio of 1.07 (+/−0.01 sd), reflecting a low level of spontaneous phage induction [44]. Upon induction by mitomycin C, the PtoH ratio increased to 14.56 (+/−1.18 sd). The difference of PtoH ratios between induced and control cultures (ΔPtoH ratio), calculated as the mean log_2_-fold change (l_2_fc) of PtoH ratios (Fig. 1c; see also the ‘Methods’ section: formula iii), was 3.65 (+/−0.13 sd).

**Fig. 2:**
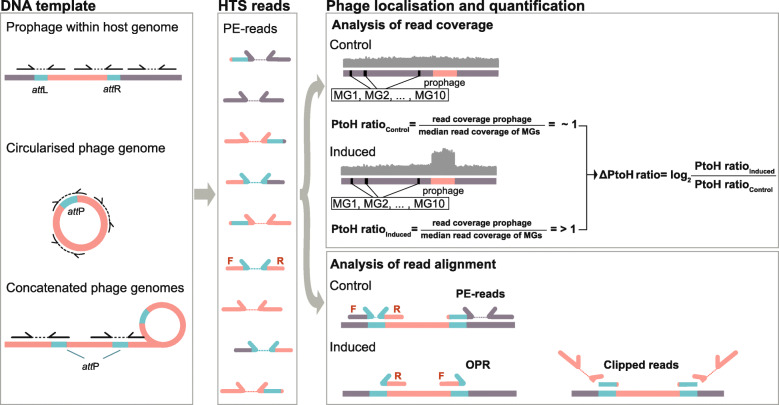
Localisation and quantification of inducible prophages by HTS reads. In our study, DNA templates from either integrated prophages (i.e. within host genomes) or induced prophages (i.e. from circularised and concatenated phage genomes) were subjected to HTS. Depending on the type of template DNA, HTS generates paired-end (PE) reads of bacterial (dark purple), viral (red) or combination of both (mixture of dark purple, turquoise and red) origin. Alignment of PE-reads from prophages to the host reference genome is expected to result in an even read coverage and a PtoH ratio around one. PE-reads from circularised and concatenated phage genomes are expected to increase the read coverage at the prophage region compared to the remainder of the reference genome resulting in a PtoH ratio larger than one. The read coverage of the reference genomes was calculated as the median read coverage of 10 universal, single-copy, phylogenetic marker genes (MGs). The ΔPtoH ratio represents the change in the prophage activity between induced and non-induced prophages measured as mean l_2_fc. Reads originating from regions containing the *att*L or *att*R sites of prophages (turquoise) reveal usual PE read characteristics (F-R orientation, standard insert size) when being aligned to the reference genome. However, PE-reads originating from regions containing the *att*P site of circularised and concatenated phage genomes (turquoise) are outward-oriented (R-F orientation) and have an enlarged insert size (approximating the genome size of the respective phages) when being aligned to reference genomes. These PE-reads are referred to as outward-oriented paired-end reads (OPRs). PE-reads originating from excised prophages and covering the *att*P site align only partially to the reference genome of the lysogen; thus, they give rise to reads we refer to as clipped reads. The accumulation of clipped reads and OPRs around *att* sites, and increased ΔPtoH ratio allow to identify and locate inducible prophages

**Fig. 3:**
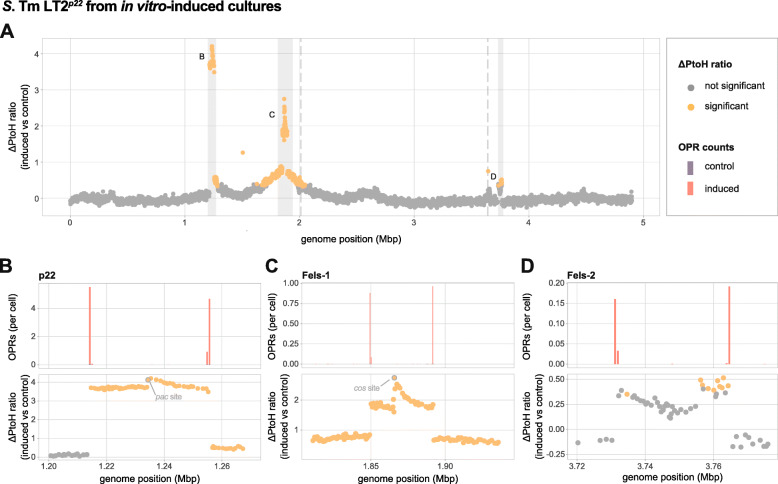
ΔPtoH ratio and replication modes of induced *S.* Tm LT2^*p22*^ prophages determined by high-throughput sequencing. (**a**) ΔPtoH ratio of each gene in mitomycin C-induced (*n*=3) *S.* Tm LT2^*p22*^ cultures shown as mean l_2_fc. The fold change in read coverage for each gene was evaluated using the DESeq2 package. Statistical significance required both a significant change of read coverage between induced and control cultures (Wald test; *p* value<0.05 after Benjamin Hochberg correction) and a l_2_fc higher than one standard deviation of the mean l_2_fc of all non-prophage genes. Data points in plots represent all protein-coding *S.* Tm LT2^*p22*^ genes and are shown in orange if the l_2_fcs were statistically significant (otherwise in grey). The l_2_fcs along the *S.* Tm LT2^*p22*^ genome resemble a one-sided and a two-sided tent-like shape around the genomic locations of the phages p22 (1,213,987-1,255,756) and FELS-1 (1,849,458-1,892,188). The grey dashed lines indicate the locations of the prophages Gifsy-2 and Gifsy-1. Detailed views of the genomic locations of the prophages p22 (**b**), Fels-1 (**c**) and Fels-2 (**d**). Mean OPR counts in induced (red) and control (dark purple) cultures are shown in the upper panel. Each bar represents the normalised mean count of OPRs that were aligned within the shown regions. The individual OPR counts per sample were normalised by the sample-specific median insert coverage of 10 single-copy MGs to yield OPR counts per cell before the overall mean OPR counts was calculated. The lower panel shows the patterns of the ΔPtoH ratio for each prophage and adjacent host regions. Orange data points with grey borders denote the genes containing the packaging site *pac* for p22 and *cos* for Fels-1

Next, we sought to compare PtoH values based on shotgun-sequenced short insert DNA libraries, from the same samples to the reference values established by qPCR (see the ‘Methods’ section). To this end, we aligned paired-end (PE) reads from control and induced cultures to a re-sequenced genome (Fig. 2) of *S*. Tm LT2^*p22*^ (see Material, Additional file 1: Supplementary Figure 1). The mean PtoH ratio for p22 [1.14 (+/−0.04 sd)], calculated as the fragment coverage of the p22 region divided by the median fragment coverage of 10 universal, single-copy, phylogenetic marker genes [45, 46] across replicates, was similar to the PtoH ratio of p22 measured by qPCR. In mitomycin C-induced cultures, the PtoH ratio increased to 14.94 (+/−1.73 sd) and the ΔPtoH ratio (Fig. 3a/b) was 3.71 (+/−0.19 sd). The PtoH ratios of p22 measured by qPCR and HTS for both control (*p*=0.10, Welch test; *p*=0.19 paired *t* test) and induced (*p*=0.77, Welch test; *p*=0.40 paired *t* test) *S*. Tm LT2^*p22*^ cultures showed no significant difference between the two methods regardless of assuming equal (paired *t* test) or unequal (Welch test) variances between the groups. The same was true for the ΔPtoH ratio between induced and control cultures (*p*=0.74, Welch test; 0.56 paired *t* test) when measured by qPCR or HTS. The close agreement with the measured qPCR values, therefore, validates our HTS method to determine accurate PtoH ratios.

To determine whether mitomycin C induces prophages other than p22, we tested for significant ΔPtoH ratios as mean l_2_fc of all genes in *S*. Tm LT2^*p22*^ using the DESeq2 package [47]. In addition to significantly increased (*p*<0.05) l_2_fcs for each gene in p22 [mean=3.76 (+/−0.13 sd)], we identified Fels-1 as a second induced prophage (Fig. 3a) with a significantly (*p*<0.05) increased mean l_2_fc of 1.93 (+/−0.22 sd). This result is in accordance with previous findings demonstrating the inducibility of Fels-1 by mitomycin C [48]. We also detected some genes within the region of Fels-2 with significant l_2_fc (Fig. 3a). However, no significant ΔPtoH ratio was observed for either Gifsy-1 or Gifsy-2, which is in accordance with a lack of virion production of Gifsy-1 and Gifsy-2 after mitomycin C-treatment in Frey et al. (2005). This could be due to mitomycin C having reduced efficiency in Gifsy-1 and Gifsy-2 induction compared to other substances like hydrogen peroxide and/or that the presence of Gifsy-1 may decrease the efficiency of Gifsy-2 induction in *S.* Tm LT2 strains [49, 50].

### Outward-oriented and clipped sequencing reads reveal genomic locations of phages in *S*. Tm LT2^p22^

The products of sequencing DNA libraries that contain circularised or concatenated phage genomes, as expected in induced cultures of *S.* Tm LT2^*p22*^, should be composed of PE reads and abnormally distant, outward-oriented PE reads (OPRs) when aligned to the reference genome of the lysogenic host (Fig. 2). This expectation is in accordance with in vitro molecular biology protocols that are used to construct circularised ‘mate-pair’ DNA libraries of genomic fragments several kb in size. In this case, the identification of OPRs in reference genome alignments provides information about the original location of the sequenced constructs. Thus, we expected that for induced prophages following this mode of replication, OPRs would accumulate at the attachment sites of prophages (*att*L/R) and that once corroborated, identifying OPRs would be a suitable approach to experimentally validate the presence of inducible prophages. In addition, sequencing reads that overlap with the beginning or the end of homologous *att* core sequences in lytic phages are expected to be only partially aligned (i.e. clipped) at the corresponding *att*R and *att*L sites in the reference genome (Fig. 2). Inspecting these clipped reads should, therefore, allow for identifying the recombining *att* core sequences of p22 [51], and thus, the exact genomic coordinates of p22 and other inducible prophages.

In line with these expectations, we observed that mitomycin C-induction led to an increased count of OPRs per cell around the respective *att*L and *att*R sites of p22, Fels-1 and Fels-2 (Fig. 3b-d). For all three phages, similar counts at the *att*R and *att*L sites provided strong evidence that these OPRs originated from intra-cellular circularised or concatenated phage genomes or mature virions (Additional file 2: Supplementary Table S1). By inspecting clipped reads, we mapped the coordinates of the p22 prophage in *S.* Tm LT2^p22^ to range from 1,213,987 to 1,255,756. Furthermore, we found the homologous core sequence of the *att* site to be in perfect agreement to the one reported initially [51]. When applying the same approach to Fels-1 and Fels-2, we mapped the genomic coordinates of Fels-1 to range from 1,849,458 to 1,892,188 and verified by clipped read analysis its 8 bp long *att* core sequence, TCCTTTCA [52]. The coordinates of Fels-2 range from 3,731,215 to 3,764,954 and we identified a 47 bp long *att* core sequence (Additional file 2: Supplementary Table S3).

### Different replication modes are reflected in distinct shapes of read coverage within and adjacent to prophage locations

In addition to analysing OPRs and clipped reads, we observed characteristic patterns within and adjacent to the genomic locations of p22 and Fels-1 in mitomycin C-induced cultures. For example, when comparing control and induced cultures, a stepwise increase in sequenced DNA fragments was observed at the *pac* site of p22, followed by a gradual decrease towards the *att*R site (Fig. 3b). The higher coverage of DNA sequencing reads downstream of the *pac* site is an expected result of the headful packaging mechanism, which leads to some redundancy of the phage genome within procapsids [43]. Moreover, we observed a significant increase in read coverage of bacterial host genes downstream of the *att*R site, resembling a one-sided tent when plotting read coverage against genome position (Fig. 3a). This pattern is likely the result of delayed prophage excision (relative to packaging initiation) resulting in unidirectional headful packaging of integrated viral and adjacent host DNA, which has recently been described as lateral transduction [53]. For Fels-1, we observed a similar stepwise increase within the prophage region, only this time located downstream of a *cos* site (Fig. 3c). In contrast to p22, however, host gene DNA fragments were significantly increased both up- and downstream of the *att*L and *att*R sites, respectively, resulting in a characteristic two-sided tent-like shape in the corresponding coverage plot (Fig. 3a). This pattern is likely a result of ‘escape replication’, during which DNA is bi-directionally amplified in situ prior to excision of Fels-1, as previously described [48]. The ‘escape replication’ could provide a fitness advantage to the phage by increasing the excision success of Fels-1 or to enhance the change of gene exchange between Fels-1 and Gifsy-2 that is replicated along with Fels-1. Notably, Frye et al. (2005) detected no adjacent host genes in Fels-1 phage particles isolated from mitomycin C-induced *S*. Tm LT2 cultures, which is expected upon excision and *cos* site-mediated unit-length packaging of phage procapsids [54]. However, the observed stepwise increase in read coverage downstream of the cos site may rather indicate that immature virions protecting the viral DNA from degradation were produced during escape replication as previously described in excision-defective λ prophages [55].

### OPRs and clipped reads reveal inducible murine gut bacterial prophages

Having validated our HTS-based methodology to assess the inducibility of prophages, to quantify PtoH ratios and to map the genomic location of prophages, we sought to apply this approach to investigate previously uncharacterised gastrointestinal prophages. Specifically, we chose to study a set of 12 gut bacterial strains, known as Oligo-MM^12^ [also termed sDMDMm2 (Additional file 2: Supplementary Table S2)], that serve as a representative, defined and experimentally tractable gut microbiota in mice [56, 57]. Although genome sequences have already been generated, the presence, inducibility and putative replication modes of prophages in these strains have not been characterised to date. In addition, we analysed a human commensal strain, *Escherichia coli* HS (*E. coli* HS) [58], one of the predominant facultative anaerobic organisms in the gastrointestinal tract [59].

**Fig. 4:**
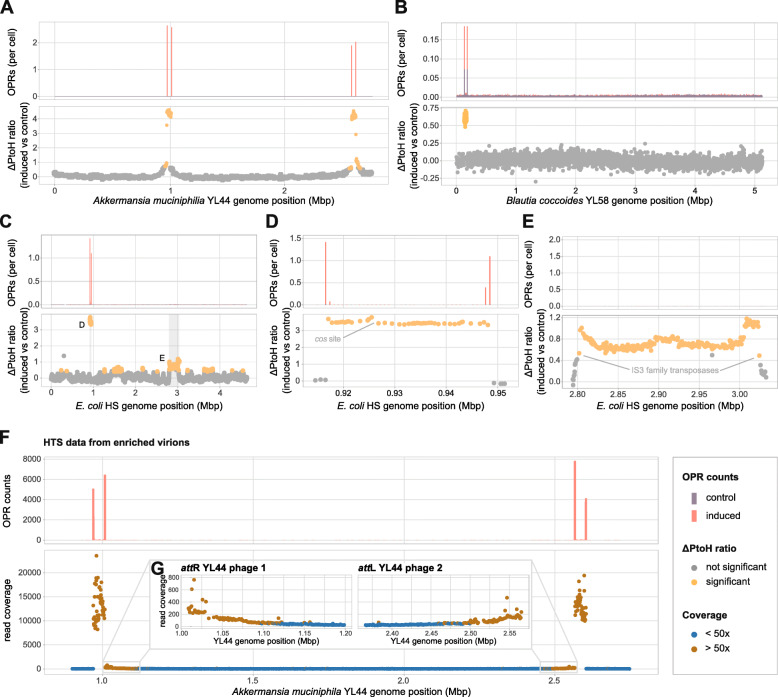
Identification of inducible prophages in murine gut model microbiota. (**a**) Mean OPR counts (upper panel) and ΔPtoH ratio shown as mean l_2_fc (lower panel) of each gene in mitomycin C-induced versus control *Akkermansia muciniphila* YL44 revealed two inducible prophages. For both prophages, a two-sided tent-like shape is observed when plotting l_2_fcs of ΔPtoH ratio for each gene against the corresponding genome position. (**b**) Same analysis as in (**a**) for *Blautia coccoides* YL58 revealed one inducible prophage. The elevated mean OPR counts in control cultures (purple bars) suggest that spontaneous induction of this prophage occurred also under control conditions. (**c**-**e**) For *E. coli* HS cultures two regions with significant l_2_fcs (orange dots) were observed. Grey areas highlight the regions shown in detail in panels **d** and **e**. Mean OPR counts were increased after induction (red) in the first (**d**), but not in the second region (**e**). The first and last gene with a significant l_2_fc are homologous to IS3 family transposases, suggesting that the second region may represent a linear mobile genetic element. (**f**) Enriched *A. muciniphila* YL44 virion DNA was sequenced to verify mature virion production upon induction, as indicated by a clear increase in OPR counts and read coverage in the prophage region. (**g**) Genes at the *att*R site of YL44 phage 1 and at the *att*L site of YL44 phage 2 showed a higher read coverage (brown circles; coverage > 50X) than other host genes (blue circles, coverage < 50X), suggesting that these phages may mediate lateral transduction. Statistical significance of l_2_fc between induced (*n*=3) and control (*n*=3) cultures was tested as described in the legend of Fig. 3. Genes with a significant fold change are shown as orange data points; non-significant ones are shown in grey

We aimed to induce putative prophages in cultures of Oligo-MM^12^ strains during the logarithmic growth phase with mitomycin C (Additional file 1: Supplementary Figure S2). To identify inducible prophages, we determined the location of OPRs and tested if the ΔPtoH ratio of the genes between OPR had a significant (*p*<0.05) mean l_2_fc. We identified two inducible prophages in *Akkermansia muciniphila* YL44 (YL44), one in *Blautia coccoides* YL58 (YL58), and another one in *E. coli* HS (Fig. 4a-c). By analysing clipped read locations, we determined the genomic coordinates for each of the identified prophages (Table 1) and used them as anchor points to identify the homologous core sequence (Additional file 2: Supplementary Table S3,). For YL44, we detected a significant (*p*<0.05) ΔPtoH ratio of 4.41 (+/−0.13 sd) for the first prophage and a significant ΔPtoH ratio of 4.19 (+/−0.14 sd) for the second prophage (Fig. 4a). To verify the production of mature virions, we enriched mitomycin C-induced YL44 cultures for viral particles, sequenced the extracted DNA (see the ‘Methods’ section), and validated the predicted start and end sites of the inducible prophages by aligning the assembled phage genomes (Additional file 3) to their host genome as well as by clipped read analysis. Although spontaneous induction was observed in control cultures of YL58, we still detected a significant (*p*<0.05) ΔPtoH ratio of 0.61 (+/−0.05 sd) per cell upon mitomycin C induction (Fig. 4b). For *Acutalibacter muris* KB18, we observed one and for and *Flavonifractor plautii* YL31 two additional OPR-flanked putative prophage region (Table 1). However, ΔPtoH ratios were not statistically significant (Additional file 1: Supplementary Figure S3), since all of these prophages were detected not only in induced but also in control samples. In the case of *E. coli* HS, we identified an inducible prophage with a *cos* site (Fig. 4d; see the ‘Methods’ section; Additional file 2: Supplementary Table S4). In addition to a significant ΔPtoH ratio of 3.51 (+/−0.13 sd) in the prophage region (Fig. 4d), which was validated by qPCR (3.53 +/−0.16 sd), a second region of 229,846 bp length showed a significantly (*p*<0.05) increased ΔPtoH ratio of 0.73 (+/−0.14 sd). However, whereas prophage induction results in an increase in OPRs around the attachment sites of the prophage, no OPR accumulation was observed for the second region (Fig. 4e). Functional annotation of the genes in this region revealed two IS3 family transposases at the start and end of the region.

**Table 1:** Clipped read analysis enables locating coordinates of inducible prophages in gut bacterial genomes

Strain	Phage	Start site prophage	End site prophage
*A. muciniphila* YL44	YL44 phage 1	969482	1008291
	YL44 phage 2	2566025	2602918
*B. coccoides* YL58	YL58 phage 1	128601	174693
*E. coli* HS	*E. coli* HS phage 1	916812	948289
*A. muris KB18*	KB18 phage 1	2602856	2642096
*F. plautii* YL31	YL31 phage 1	1009760	1046747
	YL31 phage 2	2984604	3026727

Reads originating from circularised or concatenated viral genomes align only partially to the reference genome generating clipped reads. The accumulation of clipped reads at the *attL* and *attR* sites allows for identifying the exact start and end coordinates of inducible prophages

The read coverage patterns of the newly identified phages in YL44 resembled those we previously observed for *S*. Tm LT2^*p22*^ Fels-1, that is, a two-sided tent suggesting bi-directional escape replication and concomitant production of virions (Fig. 4a). Alignment of sequencing reads from enriched virions to the reference genome of YL44 showed a substantial coverage increase at the locations of both prophages. Together with the accumulation of OPRs at the respective *att* sites (Fig. 4f), these results suggest that the sequenced DNA originated from mature virions. Notably, read coverages were increased upstream of YL44 phage 1 and downstream of YL44 phage 2, indicating a *pac* site-mediated lateral transduction potential of both phages (Fig. 4g). These results were corroborated using the software PhageTerm [60] to predict the replication mode of these phages. By contrast, for the induced phage in YL58, we observed a sharp coverage increase (Fig. 4b), similar to the one observed for *E. coli* HS; however, an accumulation of read start sites, as expected to originate from *cos* sites with a 5′-cohesive end in linear phage genomes, could not be readily identified.

### Gut prophages are inducible in ex vivo-cultured murine gut bacterial community samples

**Fig. 5:**
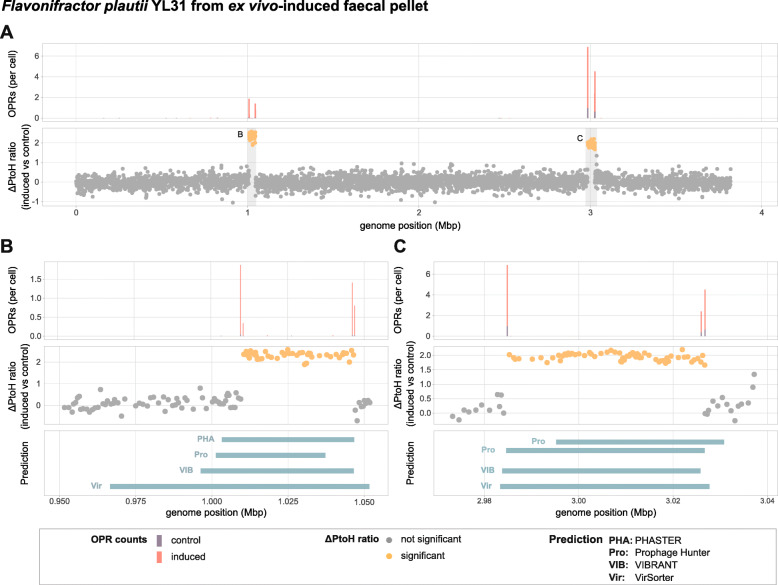
Detection of inducible prophages in ex vivo*-*cultured murine faecal samples. (**a**) Mean OPR counts (upper panel) and ΔPtoH ratio shown as l_2_fc of genes in the *Flavonifractor plautii* YL31 genome (lower panel) from  mitomycin C-induced versus control cultures of murine, faecal pellets reveal two inducible prophages. The areas highlighted in grey indicate genomic regions of prophage locations that are shown in panels **b** and **c**. (**b**/**c**) YL31 phage 1 and YL31 phage 2 show mean OPR counts (upper panels), ΔPtoH ratio (middle panels) and in silico-predicted regions of prophages in the reference genome of *F. plautii* YL31 (lower panel) using Prophage Hunter, PHASTER, VIBRANT and VirSorter. Visualisations include prophage predictions of category 1 and 2 for VirSorter, and prophages predicted to be intact or active for PHASTER and Prophage Hunter and predicted prophage of VIBRANT, respectively. None of the prediction tools was able to predict unambiguously start and end coordinates of induced prophages. Statistical significance of differences between induced (*n*=3) and control (*n*=3) cultures of *F. plautii* YL31 (**a**-**c**) was tested as described in the legend of Fig. 3

**Fig. 6:**
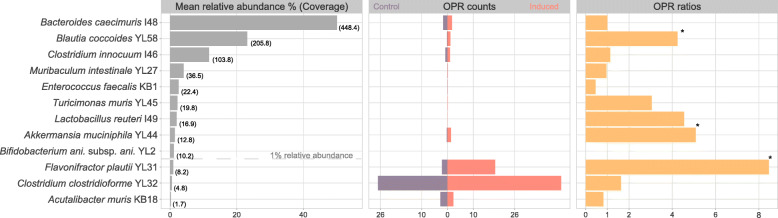
Relative abundance and OPR counts for strains in ex vivo-cultured murine faecal samples. Mean relative abundance (left panel) of each strain in murine faecal samples (*n*=6) after ex vivo culturing. The numbers within brackets denote the corresponding median per base sequence coverage of each strain. The dashed line represents the 1% relative abundance threshold. OPR counts (middle panel) over the genome of control (*n*=3, dark purple) and induced (*n*=3, red) samples, excluding OPRs at the beginning and end of the genome. The OPR counts of each sample were normalised per cell. OPR ratios (right panel) between induced and control samples. Strains, for which an active prophage was identified by ΔPtoH ratio and OPRs, are marked by an asterisk

Having experimentally validated inducible prophages in pure cultures of Oligo-MM^12^ strains, we investigated the inducibility of prophages from the intestinal community of gnotobiotic Oligo-MM^12^ mice. We evaluated whether the HTS-based approach could detect prophage induction in ex vivo-cultured communities and thus help to evaluate several strains simultaneously. We used mitomycin C to induce faecal pellets resuspended in media and performed metagenomic sequencing (see the ‘Methods’ section). By aligning PE reads to the collection of the respective reference genomes, we located OPRs and could verify that the in vitro inducible prophages of YL44 and YL58 produced circular phage genomes when induced in a community setting (Additional file 1: Supplementary Figure S4). In addition to the OPRs detected for the putative prophages in *F. plautii* YL31 identified by in vitro induction (Additional file 1: Supplementary Figure S3), we detected a significant (*p*<0.05) ΔPtoH ratio of 2.31 (+/−0.16 sd) for YL31 phage 1 and 1.95 (+/−0.11 sd) for YL31 phage 2 (Fig. 5a-c) through ex vivo*-*induction. Clipped read inspection specified genomic ranges from 1,009,760 to 1,046,747 (YL31 phage 1) and from 2,984,604 to 3,026,727 (YL31 phage 2). The differential inducibility of YL31 phages between in vitro and ex vivo conditions may be due to an indirect community response to mitomycin C, or other factors, such as nutrient limitation [61] activating the lytic cycle during ex vivo culturing. We found that our method detected inducible prophages not only in highly abundant strains (*Blautia coccoides* YL58) but also strains that were present at low relative abundance (*A. muciniphila* YL44: 1.4%, *F. plautii* YL31: 0.9%). Notably, we detected a high number of OPRs within one region of a strain (*Clostridium clostridioforme* YL32) with a relative abundance and sequence coverage of as low as 0.55% and 4.8, respectively (Fig. 6). Despite this evidence for the detection of an additional putative prophage, we did not follow up on this region due to our conservative requirement of a statistically significant ΔPtoH ratio in the same region.

### In silico predictions of prophage locations are variable and disagree between tools

Several in silico tools are available to predict prophages in microbial genomes and metagenomic assemblies [34–37], which have been used to expand the number of known prophages, especially in bacteria that are challenging to be cultured. Nevertheless, most prediction tools do not account for mutations or biological interactions affecting prophage activity. Therefore, the validity of in silico predictions requires experimental validation to assess the precision in predicting the genomic location and the inducibility of putative prophages. We sought to assess several prediction tools (Additional file 4; Supplementary Table S5-8), for their ability to detect inducible prophages and their accuracy of prophage localisation in reference genomes by comparing their output to our experimentally validated data.

For most predicted prophages (>90%), we could not verify their inducibility using a sub-lethal concentration of mitomycin C. It is not possible to ascertain whether these prophages were not induced under the specific experimental conditions of our study or whether they cannot be induced at all (e.g. inactive/cryptic prophages). However, for those prophages that were induced in our study in vitro or ex vivo, we found that the predicted locations were in poor agreement with our experimental data as well as inconsistent across the tested tools. For example, Prophage Hunter and VirSorter predicted two alternative prophage locations for YL44 phage 1, which were additionally different between the tools and entirely missed by PHASTER (Table 2). We also sought to obtain a more quantitative measure for tool performance by scoring the precision and sensitivity of the various tools (Table 2) for prophage localisation. As a result, we found that no specific tool outperformed the others. For example, even though Prophage Hunter was the only tool that was able to correctly predict the start and end for a prophage (YL31 phage 2); it also predicted a second possible start and end site for the same prophage (Fig. 5c). VirSorter was the only tool that predicted every prophage, although for two predicted prophages (*E. coli* phage 1 and p22) it had an equal score with PHASTER (Table 2).

**Table 2:** Performance evaluation of different tools to predict prophage locations (start/end sites)

Experimental data	Prediction				Evaluation		
Prophage (range/length—including *att* sites)	Tool	Deviation from		Length	Precision	Recall	F-score
		Start	End				
*S.* Tm LT2^*p22*^ p22 (1213987-1255756/41770)	PHASTER	60	1126	42836	0.97	1.00	0.99
	Prophage Hunter	−13077	8273	63120	0.66	1.00	0.80
	**VIBRANT**	**60**	−**334**	**41376**	**1.00**	**0.99**	**1.00**
	Virsorter	10	1152	42912	0.97	1.00	0.99
*S*. Tm LT2^*p22*^ Fels-1 (1849458-1892188/42731)	PHASTER	−1528	−3284	40975	0.96	0.92	0.94
	Prophage Hunter	−4528	7083	54342	0.79	1.00	0.88
	**VIBRANT**	**41**	**1216**	**43906**	**0.97**	**1.00**	**0.99**
	Virsorter	−20661	22091	85483	0.50	1.00	0.67
*S*. Tm LT2^*p22*^ Fels-2 (3731215-3764954/33740)	**PHASTER**	**0**	**1410**	**35150**	**0.96**	**1.00**	**0.98**
	Prophage Hunter	NA	NA	NA	NA	NA	NA
	VIBRANT	449	10659	43950	0.76	0.99	0.86
	Virsorter	−115370	79199	228309	0.15	1.00	0.26
YL44 phage 1 (969482-1008291/38810)	PHASTER	NA	NA	NA	NA	NA	NA
	Prophage Hunter	−9199	−2567	45442	0.80	0.93	0.86
	Prophage Hunter	23847	3354	18317	0.82	0.39	0.52
	VIBRANT	186	−9968	28656	1.00	0.74	0.85
	**Virsorter**	**1436**	**138**	**37512**	**1.00**	**0.96**	**0.98**
	Virsorter	8087	138	30861	1.00	0.79	0.88
YL44 phage 2 (2566025-2602918/36894)	PHASTER	NA	NA	NA	NA	NA	NA
	Prophage Hunter	−4041	−23277	17658	0.77	0.37	0.50
	**Prophage Hunter**	**704**	−**2995**	**33195**	**1.00**	**0.90**	**0.95**
	Prophage Hunter	7638	10625	39881	0.73	0.79	0.76
	VIBRANT	−10070	−3356	43608	0.77	0.91	0.83
	Virsorter	−25233	44693	106820	0.35	1.00	0.51
YL58 phage 1 (128601-174693/46093)	PHASTER	NA	NA	NA	NA	NA	NA
	Prophage Hunter	−2929	−27047	21975	0.87	0.41	0.56
	**Prophage Hunter**	**139**	−**49**	**45905**	**1.00**	**1.00**	**1.00**
	VIBRANT	153	3341	49281	0.93	1.00	0.96
	Virsorter	−1242	33898	81233	0.57	1.00	0.72
*E. coli* HS phage 1 (916812-948289/31478)	**PHASTER**	**78**	**3104**	**34504**	**0.91**	**1.00**	**0.95**
	Prophage Hunter	NA	NA	NA	NA	NA	NA
	VIBRANT	461	4880	35897	0.86	0.99	0.92
	**Virsorter**	**2787**	−**41**	**28650**	**1.00**	**0.91**	**0.95**
YL31 phage 1 (1009760-1046747/36988)	**PHASTER**	−**6510**	−**17**	**43481**	**0.85**	**1.00**	**0.92**
	Prophage Hunter	−8493	−9478	36003	0.76	0.74	0.75
	VIBRANT	−13474	−185	50277	0.73	0.99	0.84
	Virsorter	−43160	4955	85103	0.43	1.00	0.61
YL31 phage 2 (2984604-3026727/42124)	PHASTER	NA	NA	NA	NA	NA	NA
	**Prophage Hunter**	**0**	**0**	**42124**	**1.00**	**1.00**	**1.00**
	Prophage Hunter	10596	4114	35642	0.88	0.75	0.81
	VIBRANT	−828	−914	42038	0.98	0.98	0.98
	Virsorter	−1257	983	44364	0.95	1.00	0.97
KB18 phage 1 (2602856-2642096/39241)	**PHASTER**	−**5132**	**7663**	**52036**	**0.75**	**1.00**	**0.86**
	Prophage Hunter	3156	15738	51823	0.70	0.92	0.79
	VIBRANT	NA	NA	NA	NA	NA	NA
	Virsorter	−45270	5505	90016	0.44	1.00	0.61

The accuracy of prediction tools to locate prophages was evaluated by comparing the predicted start and end coordinates to the corresponding data obtained by experimental validation. The assessment includes the recall, precision and F-score of correctly predicting prophage genomic coordinates. The first column reports the range and length of each prophage based on experimental data as determined by analysis of clipped reads. Columns two to five summarise the results of the different prediction tools. The deviations from the validated start and end positions are shown with negative and positive numbers if the predicted site was upstream or downstream of the true start or end site, respectively. For each prophage, the tool with the best F-score is highlighted in bold. In the case of a tie, both tools with the highest F-score are highlighted. Prophages that were not categorised as ‘active’ (Prophage Hunter), ‘complete’ (PHASTER), ‘category 1’ or ‘category 2’ (VirSorter), or not predicted at all, are reported as NA

## Discussion

The activity of prophages in host bacteria has previously been studied either by qPCR [62, 63] or aligning HTS reads from experimentally enriched virions or faecal samples to reference genomes of lysogens [27, 29, 33]. However, to our knowledge, this is the first time that PtoH ratios derived from HTS data have been experimentally validated against those obtained by qPCR measurements. Although associated with higher costs, one major advantage of the HTS-based approach is that the relative activity of induced prophages can be quantified without prior knowledge of the location of *att* sites in the host genome. As shown here for several bacterial strains from a defined gut microbiota collection, the location of inducible prophages that form circularised intermediate products during replication can be approximated by identifying OPRs and precisely determined by inspecting clipped read alignments. However, our results also show that verification of the presence of viral hallmark genes is needed to distinguish prophages from other circularising mobile genetic elements that may also be transferred as dsDNA [64, 65].

We found that both the availability of appropriate reference genomes (Additional file 1; Supplementary Figure S1) and experimental validation of prophage regions can be beneficial for an accurate quantification of PtoH ratios. The demonstrated applicability of the OPR-based approach to ex vivo-induced faecal samples and the subsequent localisation of a prophage within a strain with less than 5x sequence coverage highlights the potential of the presented approach to study inducible prophages of microbiota members that are present at low relative abundances, such as *F. plautii* YL31 in the Oligo-MM^12^ model [56]. However, low sequence coverage may not allow for the detection of specific replication patterns, and it should be noted that PtoH ratios measured in cultures of individual strains or communities only represent average values for a sampled population. To study the burst size of prophages or to resolve spatio-temporal heterogeneities in prophage activity within microbial populations will require determining these ratios at single-cell resolution.

Our results on comparing the performance of a selection of prophage prediction tools revealed that experimental validation is currently unmatched in terms of accuracy for determining the location of prophages, even of known ones, within bacterial host genomes. Furthermore, in our analyses, only a fraction of the predicted prophages were inducible using a single stimulus. It may thus be possible that prediction tools overestimate the number of inducible prophages by detecting and mislabelling cryptic prophages, which may constitute a sizable fraction of a species’ accessory genome [66]. However, the presented methods are restricted to locate complete, host-integrated, lysogenic prophages with circularised and/or concatenated genomes produced upon their induction and excision. Thus, other predicted prophages, despite being inducible, may not have responded to the experimental conditions tested here. In future studies, the use of an extended spectrum of induction stimuli may help to improve our knowledge about inducible, circularising prophages in a variety of bacteria, and help to populate virus-centric databases and to improve the accuracy of bioinformatic prediction tools.

## Conclusions

To improve our understanding of phage-microbe interactions and their relevance in mammalian health and disease, it is critical to overcome current challenges in locating inducible prophages and in quantifying their activity in gut microbial communities. The present work demonstrates the suitability of experimental induction and subsequent analysis of HTS data as a powerful approach to tackle these challenges using gut microbial strains cultured either individually or as communities starting from mouse faecal samples. Experimental validation of predicted prophages remains an important task for the annotation of bacterial genomes, given that predictions from bioinformatics tools alone may be incongruent and insufficiently accurate.

## Methods

### Induction and extraction of *Salmonella enterica* serovar Typhimurium LT2^*p22*^

*Salmonella enterica* serovar Typhimurium LT2 carrying a lysogenic p22 phage (*S*. Tm LT2^*p22*^) was cultured overnight in lysogeny broth (LB; 10 g L^−1^ tryptone (Oxoid), 5 g L^−1^ yeast extract (Oxoid), 5 g L^−1^ NaCl (Thermo Fisher)) supplemented with 1.23 g L^−1^ MgSO_4_·7H_2_O (Sigma-Fluka) before it was diluted 1:20, subcultured for 4 h and further diluted to reach a final concentration of 10^5^ bacteria mL^−1^. We separated the cultures into control and induced cultures. The induced cultures were supplemented with 1 μg mL^−1^ mitomycin C (Sigma-Aldrich), and all cultures were aerobically incubated for 24 h at 37 °C and shaken at 180 min^−1^ (Kühner Shaker). We used 100 μL of each culture for DNA extraction using the AllPrep DNA/RNA Kit (Qiagen) with the following modification in the disruption step: the samples were mixed with 0.6 mL of RLT buffer complemented with 10 μL 2-beta-mercaptoethanol (Sigma-Aldrich), added to 2 mL tubes pre-filled with 100 μm Zirconium beads (OPS Diagnostics LLC) and disrupted twice at 30 Hz for 3 min with 5 min incubation between each disruption step using the mixer mill Retsch MM400. Samples were centrifuged at full speed for 1 min to pellet the cell debris and then transferred to DNA-binding columns. We eluted the DNA in 60 μL elution buffer (EB) and used it as templates for qPCRs and high-throughput sequencing. Library preparation using the TruSeq Nano DNA Library Kit (Illumina) and sequencing was carried out by the Functional Genomics Centre Zurich using the NovaSeq 6000 platform and 2×150 bp PE-reads with a target insert size of 500 bp. For each sample, the output was on average 6.9 M reads.

### In vitro induction of murine intestinal model bacterial strains

Oligo-MM^12^ strains [56, 57] were individually cultured for 12-24 h in supplemented brain heart infusion medium (BHIS) (37 g L^−1^ brain heart infusion broth (Thermo Fisher), 2.5 mg L^−1^ hemin (Sigma-Aldrich), 1.23 g L^−1^ MgSO_4 _· 7 H_2_O (Sigma-Fluka), 2 g L^−1^ NaHCO_3_ (Sigma-Aldrich), 500 mg L^−1^ L-cysteine (Sigma-Aldrich), 500 μg L^−1^ menadione (Sigma-Aldrich)). The two exceptions were *Akkermansia muciniphila* YL44 for which the BHIS was additionally supplemented with 25 mg L^−1^ mucin (Sigma-Aldrich) and *Lactobacillus reuteri* I49, which was cultured in 51 g L^−1^ MRS broth supplemented with 1 mL L^−1^ Tween 80 and 1.23 g L^−1^ MgSO_4_·7H_2_O (Sigma-Fluka). The cultures were diluted 10^−1^ to the final volume of 6 mL and sub-cultured for 1 to 15 h to reach the logarithmic growth phase before each culture was divided equally into control and induced cultures. The induced cultures were supplemented with 1 μg mL^−1^ mitomycin C (Sigma-Aldrich) and both cultures were incubated at 37 °C for 1-3 days. *Escherichia coli* HS was cultured as described for *S.* Tm LT2^*p22*^ with the exception of using BHIS instead of LB. All the handling and culturing of the Oligo-MM^12^ and *E. coli* HS strains were carried out in an anoxic atmosphere (7% H_2_, 10% CO_2_, rest N_2_). Afterwards, the cultures were transferred to Amicon tubes (MWCO 10,000), centrifuged at 3400×*g* for 30 min and washed with SM buffer (5.8 g L^−1^ NaCl, 2g L^−1^ MgSO_4_·7H_2_O and 50 mM L^−1^ Tris-HCl pH 7.4) before finally being concentrated to 200 μL. For additional cell lysis, the concentrate was mixed with 174 μL TE-buffer (30 mM Tris-HCl and 1 mM EDTA) supplemented with 30 mg mL^−1^ Lysozyme (Sigma-Aldrich), 1.6 U mL^−1^ Proteinase K (NEW ENGLAND BioLabs) and 10 U mL^−1^ Mutanolysin (Sigma-Aldrich) and incubated at 37 °C for 30 min. The DNA was finally extracted following the same protocol used for *S*. Tm LT2^*p22*^ with the adjustment that the volume of RLT-buffer was increased to 0.75 mL. Library preparation and sequencing was carried out by Novogene using the NEBNext DNA Library Prep Kit with a target insert size of 350 bp and using the NovaSeq 6000 platform with 2×150 bp PE-reads, respectively. For each sample, the output was on average 10.2 M reads.

### Ex vivo induction of murine gut bacterial community samples

We collected the fresh faecal pellets from three co-housed mice and separated them into halves for control and induced cultures. We homogenised each half in 0.5 mL anoxic BHIS medium containing one 3 mm metal bead at 10 Hz for 30 using the mixer mill Retsch MM400. The homogenised cultures were transferred into Hungate tubes containing 5 mL prewarmed anoxic BHIS medium. The induced cultures were treated with 1 μg mL^−1^ mitomycin C and anaerobically cultured with the control samples at 37 °C, shaken at 180 min^−1^ (Kühner Shaker) for 6 h. From each culture, 200 μL were pretreated with 174 μL TE-buffer (30 mM Tris-HCl and 1 mM EDTA) supplemented with 30 mg mL^−1^ Lysozyme (Sigma-Aldrich), 1.6 U mL^−1^ Proteinase K (NEW ENGLAND BioLabs) and 10 U mL^−1^ Mutanolysin (Sigma-Aldrich) and incubated at 37 °C for 30 min before the DNA was extracted as described above for *S*. Tm LT2^*p22*^. The library preparation using TruSeq Nano DNA Library Kit (Illumina) and the sequencing was carried out by the Functional Genomics Centre Zurich using the NovaSeq 6000 platform and 2×150 bp PE-reads with a target insert size of 500 bp. For each sample, the output was on average 28.2 M reads.

### *Akkermansia muciniphil*a YL44 virion enrichment and sequencing

For the extraction of free *Akkermansia muciniphil*a YL44 virions, 1 L of axonic BHIS medium supplemented with 25 mg L^−1^ mucin (Sigma-Aldrich) was inoculated with 10^6^ cells mL^−1^ of *Akkermansia muciniphil*a YL44 and sub-cultured for 11 h at 37 °C before induction with mitomycin C (1 μg mL^−1^). After 48 h of induction at 37 °C, the virions were separated from the bacteria by two repeated centrifugation steps at 7000×*g* for 30 min using a Sorvall RC-6 centrifuge (Thermo SCIENTIFIC) and an additional filtration step using a 0.22-μm bottle filter (Filter Top 500 (TPP)). Between the centrifugation steps, the supernatant was transferred into a new sterile bucket without disturbing the pellet. To precipitate the free virions, 1 mL of 10 g L^−1^ FeCl_3_ · 6H_2_O was added to the supernatant, mixed and incubated at room temperature for 1 h before centrifugation at 27,000×*g* for 1 h using a Sorvall LYNX 6000 centrifuge (Thermo SCIENTIFIC). Afterwards, the supernatant was removed without disturbing the pellet, and the pellet subsequently resuspended in 3 mL ascorbic-EDTA buffer (0.4 M Mg_2_-EDTA and 0.8 M ascorbic acid, pH 6-7) before concentrated using Amicon tubes (MWCO 10.000). The concentrate was washed three times using 15 mL SM buffer before it was finally concentrated to 200 μL. The concentrate was treated with 50 U mL^−1^ DNase I (Thermo SCIENTIFIC) and RNase H (NEW ENGLAND BioLabs) at 37 °C for 2 h to remove free DNA and RNA before the reaction was interrupted with 45 μL 0.5 M EDTA. Before the extraction (using the standard extraction protocol), the concentrate was incubated with 3.8 U Proteinase K at 50 °C for 30 min and centrifugation at 17,000×*g* for 3 min to remove cell debris. The DNA was eluted in 100 μL EB buffer and concentrated to 15 μL using the genomic DNA clean & concentrator kit (Zymo Research). The viral DNA was sequenced at Novogene using the NovaSeq 6000 platform and 2×150 bp PE-reads with a target insert size of 350 bp. For the virion sample, the output was 12.6 M reads.

### NanoPore re-sequencing and assembling of *Salmonella enterica* serovar Typhimurium LT2^*p22*^

*S*. Tm LT2^*p22*^ was cultured as described above with the modification that a 30 mL subculture was used for the extraction of high molecular weight DNA using the NucleoBond AXG Columns (Macherey-Nagel) and NucleoBond Buffer Set III (Macherey-Nagel) following the supplier’s protocol. As recommended, the G3 buffer was supplemented with 100 mg mL^−1^ Lysozyme (Sigma-Aldrich), and the final DNA pellet was additionally washed with 70% ethanol before it was redissolved for 1 h at 55 °C in 150 mL EB buffer (Qiagen). The DNA quality and quantity were checked by a NanoDrop photo spectrometer (260/280: 1.89 and 260/230: 2.06) and a Qubit™ 4 Fluorometer (Invitrogen), respectively, before 400 ng of DNA was used as input for library preparation (SQK-RAD004). Sequencing was performed on a MinION R9 flow cell (Oxford Nanopore Technologies) for 24 h, producing approximately 11.5 Gbp read data, with a median read length of just over 2800 bp. Reads were basecalled with Guppy-CPU (v.3.2.4+d9ed22f; Oxford Nanopore Technologies) with default parameters. Adaptors were trimmed with Porechop (v0.2.4) [67] and then filtered to keep only the highest quality 500 Mbp with Filtlong (v.0.2.0) [68]. Assembly was performed with Flye (v.2.4.1) [69] aiming for a genome size of 5 Mbp, resulting in two contigs of size 4.95 Mbp and 95 kbp. The assembly was polished with five cycles of Pilon (v.1.23) [70], using the Illumina reads, aligned with BWA mem (v.0.7.17-r1188) [71], which resulted in final contig sizes of 4.9 Mbp and 94 kbp.

### Quality control of high-throughput sequencing data

Raw sequencing data were quality controlled using the command *bbduk.sh* of BBMap (v.38.71) [72]. First, we removed adapters from the reads, followed by the removal of reads that mapped to quality control sequences (PhiX genome). We discarded low quality reads by applying the parameters *trimq=14*, *maq=20*, *maxns=1* and *minlength=45*.

### Gene abundance calculation

The quality-controlled forward and reverse reads were mapped against the respective host genome using the bwa mem (v.07.17-r1188) algorithm [71] in PE mode and using the *-a flag*. Alignments were filtered to be at least 45 bases in length, with an identity of ≥ 97% to the reference genome and covering ≥ 80% of the query sequence. The resulting alignment file was used as input for featureCounts (v.2.0.0) [73] allowing multi-overlapping reads and multi-mapping reads (*-O -M --fraction*) to retrieve gene counts. Inserts were counted instead of reads (*-p*) and incomplete inserts with only one aligned read were discarded (*-B*). The statistical analysis of differential insert counts per gene in control and induced samples was done in RStudio (v1.2.5042) with R (v.3.6.3) using the command *DEseq* from the DESeq2 (v.1.22.2) package, applying *mean* as fitType for dispersion fitting. As output, DESeq2 provided log_2_-fold change (l_2_fc) values and *p* values based on Wald tests, adjusted by the Benjamin-Hochberg procedure. For statistically significant changes between induced and control cultures, we required an adjusted *p* value<0.05 and an l_2_fc more than one standard deviation away from the mean l_2_fc of all non-prophage genes.

### Identification and localisation of prophages in lysogenic host genomes by OPRs and clipped reads

Quality controlled forward and reverse reads were mapped individually against the respective host genome using bwa mem (version 0.7.17-r1188) with the *-a flag.*. Alignments were filtered to be at least 45 bases in length, with an identity of ≥ 97% to the reference genome and covering ≥ 80% of the query sequence. OPR detection was performed with the mVIRs package (version 1.0, https://github.com/SushiLab/mVIRs). To detect OPRs, reads were grouped to inserts by name and high and low boundaries for insert sizes of properly paired reads were estimated using the mean insert sizes and ± seven standard deviations of uniquely mapped inward-oriented paired-end reads (IPRs). OPRs were detected as follows: For each insert, first, find the best scoring alignment pairs within 3% of the best alignment score. Second, report the insert as OPR if there is no IPR with reasonable insert size within the 3% cutoff and if the OPR is the best scoring alignment. To evaluate the mean OPR count per cell, we split the genome into bins with the size of the median gene length of the respective bacterial genome. For each OPR, we identified the two bins into which the forward and the reverse reads fell and calculated the midpoint of each bin used as the input for undirected graph building using the command *graph_from_data_frame* from the package igraph (v.1.2.4.2). We excluded OPR pairs with an insert size smaller than 1000 bp and larger than host genome length minus the genome length of smallest circular dsDNA bacteriophage on NCBI [74] for the graph building. We extracted the edges, which represent the OPRs, using the command *get.edgelist* and counted the edges with the same coordinates. The sum was normalised by the median coverage of 10 universal, single-copy, phylogenetic marker genes (MGs) and the bin length to evaluate the number of OPRs per cell. As the last step, we calculated the mean OPR count for each bin from the triplicates. OPRs falling into the surrounding regions of the left and right attachment site +/− three times the insert size was summed up to verify that the number of OPRs at *att*R and *att*L sites were the same.

For the precise determination of the start and end position of a prophage, the quality-controlled reads were individually mapped as described above. We sorted, indexed and filtered the output bam files for reads that have a soft clip (-S) followed by a match (-M) or reads with the reverse feature (-M, -S). The described features are expected from viral reads, covering the prophage attachment (*att*P) site in concatemeric or circularised phage genomes when mapped to the host reference genome. To determine the end position of the prophage, the reported start position of the aligned read was adjusted by adding the number of exact matched bases −1. The count of clipped reads, indicating the unique start and end position, was evaluated and the positions with the highest counts with the exception of start and end positions of contigs were deemed as indicative for prophage boundaries. The coordinates were additionally verified by cross-matching them to the regions of increased OPR counts.

### Assembly of *Akkermansia muciniphila* YL44 phage genomes from enriched virions

The paired-end DNA sequencing reads from enriched virions were aligned with BWA [71] to the YL44 genome. Reads aligning to the predicted prophage regions were downsampled to 2×2500 reads and assembled with SPAdes (version 3.14) [75] using the option --isolate. Clipped read analysis to verify start and end sites of both phages was performed as described before.

### Estimation of genome coverage for each sample

For the normalisation to per-cell counts, we estimated the genome coverage of each sample by calculating the median insert coverage of 10 universal single-copy marker genes (MGs) [45, 46]. For each strain, the 40 original marker genes were extracted using fetchMGs (v1.1) [45], and the following 10 MGs were subsampled (COG0012, COG0016, COG0018, COG0172, COG0215, COG0495, COG0525, COG0533, COG0541 and COG0552). For each sample, we calculated the median insert coverage of the 10 MGs using the insert coverage from featureCounts as input. The median insert coverage of the 10 MGs was used to normalise the OPRs count and to calculate PtoH ratios in *S.* Tm LT2^*p22*^.

### Relative abundance and coverage calculation for ex vivo community samples

The base coverage (number of sequenced nucleotides per base of a genome) for each strain was approximated as the product of the median insert coverage of the 10 MGs and the average insert length. Subsequently, the base coverage per strain was calculated as the mean over all six (three control and three induced) replicates. The relative abundance of each strain within a community sample was calculated as the insert coverage per strain divided by the total insert coverage of all strains in the community.

### Verification of p22 prophage induction and comparison of PtoH ratios from qPCR and HTS experiments

For the verification of p22 prophage induction, we designed qPCR primers flanking the 66 bp long attachment sites (*att*L and *att*R) of lysogenic p22 and its host *S*. Tm LT2^*p22*^*.* The combination of the different primers allowed for the quantification of naïve *S*. Tm LT2^*p22*^ (Sal1: 5′-CAGGGCCGATATAGCTCAGT-3′ and Sal2: 5′-TGGTGTTTTTGAGAAATGAGGTTGT-3′), lytic p22 (p1: 5′-AGGTATGACGTGGTATGTTGTTG-3′ and p2: 5′-AAGGAAGGCACGAATAATACGTAAG-3′), and lysogenic *S*. Tm LT2^*p22*^ (Sal1 and p2) genomes. We analysed DNA of induced and control cultures of *S*. Tm LT2^*p22*^ with Universal FastStart SYBR Green Master (ROX; Roche) on the StepOne Plus real-time PCR system (Applied Biosystems) using the following cycling conditions: (1) initial denaturation, 95 °C for 14 min; (2) denaturation, 94 °C for 15 s; (3) annealing, 60 °C for 30 s; (4) extension, 72 °C for 20 s. Cycles 2–4 were repeated 35 times. The prophage to host (PtoH) ratio_qPCR_ and ΔPtoH ratio was quantified by using the formula I and III, respectively. Using HTS data, it is not possible to differentiate whether phage sequences originated from excised or inserted prophages. Similarly, bacterial sequences could originate from either naïve *S*. Tm LT2^*p22*^ or lysogenic *S*. Tm LT2^*p22*^ genomes. We thus included the *S*. Tm LT2^*p22*^ term in the formula to calculate PtoH ratio_qPCR_ to allow for a direct comparison to PtoH ratio_HTS_, which was calculated according to formula II. For the calculation of the PtoH ratio_HTS_, raw sequencing reads were processed as described above and the alignment files used as input for featureCounts to retrieve gene counts. The main difference was that the prophage sequence was used as one feature (gene) to avoid possible double-counting due to reads spanning across multiple genes. We then determined the insert coverage of the non-prophage region, approximated by the median insert coverage of the 10 MGs, and the PtoH ratio_HTS_, and the ΔPtoH ratio was calculated as shown in formula II and III, respectively. We applied two statistical tests to evaluate the mean difference between PtoH ratio and ΔPtoH ratio from qPCR and HTS experiments, one accounting for different variance between groups (Welch test) and another one assuming equal variance between groups (paired *t* test).

^I)^ PtoH ratio_qPCR_ = (p22 + *S*. Tm LT2^*p22*^)/( naïve *S*. Tm LT2^*p22*^ + *S*. Tm LT2^*p22*^)

^II)^ PtoH ratio_HTS_ = insert coverage of p22/mean insert coverage of MGs

^III)^ ΔPtoH ratio_qPCR/HTS_ = log_2_ (PtoH ratio_induced_/PtoH ratio_control_)

For additional validation, the following qPCR primers pairs were used for the quantification of naïve *E. coli* HS (attL-f: 5′-TTGAGAGGGTTGCAGGGTAG-3′ and attR-r: 5′-TTGTGGAGCCCATCAACCC-3′), lysogenic *E. coli* HS (attL-f and attL-r: 5′-CCGTAATAACCACCACTGACCA-3′) and lytic *E. coli* HS phage 1 (attR-f: 5′-CGCCATTTTGTCGCCATCG-3′ and attL-r) genomes.

### In silico prophage prediction and comparison to experimentally validated data

In addition to the re-sequenced *S.* Tm LT2^*p22*^ genome, we used the reference genomes of the Oligo-MM^12^ and *E. coli* HS strains that were downloaded from NCBI (Additional file 2: Supplementary Table S2) for the in silico prediction of prophages using PHASTER [36], Prophage Hunter [37] ,VIBRANT [35] and VirSorter [34]. For the visual presentation and *F*-score calculation, we consider for PHASTER and Prophage Hunter prophages that were classified as complete or active and for VirSorter prediction in category one and two.

### Prediction of prophage replication modes by HTS data from free virions

The replication mode of induced prophages in *A. muciniphila* YL44 cultures was predicted using sequenced reads of free virions that were provided as input for PhageTerm [60]. As prophage reference genomes, we supplied the prophage sequences (Supplementary File S3) that we extracted from the host reference genome sequence using the prophage start and end coordinates determined by clipped read analysis.

### *Cos* site prediction in prophages

Prophages that follow a unit-length packaging model initiate the packaging of viral DNA into the procapsid at the same position called the *cos* site. Therefore, the number of reads starting at a *cos* site must be increased compared to any other position in the prophage genome when the DNA originated from packed, viral DNA that was randomly sheared during library preparation. As input for the *cos* site prediction, we used quality-controlled reads, which were individually mapped as described for the OPR analysis. For the *cos* site identification, we filtered for properly paired reads that fell into the respective prophage regions. The start and end positions of reads were counted as described above for the clipped read analysis and putative *cos* sites were determined by visually inspecting sites where reads with the same start and end position accumulated within prophage regions.

## Supplementary Information

**Additional file 1: Supplementary Figure S1.** A correct reference genome sequence is favourable for determining accurate ΔPtoH ratios. **Supplementary Figure S2**: Several in vitro-induced Oligo-MM^12^ strains reveal genomic regions with putative prophages. **Supplementary Figure S3.** OPR analysis localtes a putative prophage region in cultured *A. muris* KB18 and *F. plautii* YL31***.*** **Supplementary Figure S4.** Ex vivo induction of prophages in *A. muciniphila* YL44 and *B. coccoides* YL58.

**Additional file 2: Supplementary Table S1.** Similar OPR counts at the *att*L and *att*R sites verify an origin of sequencing reads from circularised phage genomes. **Supplementary Table S2.** Collection of strains and microbiota community used in this study. **Supplementary Table S3.** Homologous core sequences of the attachment sites of induced prophages. **Supplementary Table S4.** Packaging site and its location in the lysogenic host genome.

**Additional file 3. ***Blautia coccoides* YL58 phage 1. *Flavonifractor plautii* YL31 phage 1. *Flavonifractor plautii* YL31 phage 2. *Salmonella enterica* serovar Typhimurium LT2 p22. *Salmonella enterica* serovar Typhimurium LT2 FELS-1. *Salmonella enterica* serovar Typhimurium LT2 FELS-2. *Escherichia coli* HS phage 1. *Acutalibacter muris* KB18 phage 1. *Akkermansia muciniphila* YL44 phage 1. *Akkermansia muciniphila* YL44 phage 1 (assembly virion enrichment). *Akkermansia muciniphila* YL44 phage 2. *Akkermansia muciniphila* YL44 phage 2 (assembly virion enrichment)

**Additional file 4: Supplementary Table S5.** Prophage predicted by VIBRANT. **Supplementary Table S6.** Prophage predicted by VirSorter. **Supplementary Table S7.** Prophage predicted by Prophage Hunter. **Supplementary Table S8.** Prophage predicted by PHASTER

## Data Availability

The datasets generated and analysed during the current study have been deposited at the European Nucleotide Archive (ENA) with the accession no. PRJEB39818. The mVIRs (version 1.0) package for OPR detection can be found under: https://github.com/SushiLab/mVIRs. An R-Markdown for the OPR count analysis including example files is available on Zenodo 10.5281/zenodo.4310165).
