# Supplementary material for: High throughput sequencing provides exact genomic locations of inducible prophages and accurate phage-to-host ratios in gut microbial strains
Source: Microbiome. 2021 Mar 29;9:77. doi: 10.1186/s40168-021-01033-w (PMC8008629; doi:10.1186/s40168-021-01033-w)
Supplement: Supplementary file 2 — Additional file 1: Supplementary Figure S1. A correct reference genome sequence is favourable for determining accurate ΔPtoH ratios. Supplementary Figure S2: Several in vitro-induced Oligo-MM12 strains reveal genomic regions with putative prophages. Supplementary Figure S3. OPR analysis localtes a putative prophage region in cultured A. muris KB18 and F. plautii YL31. Supplementary Figure S4. Ex vivo induction of prophages in A. muciniphila YL44 and B. coccoides YL58. [file 40168_2021_1033_MOESM1_ESM.pdf]

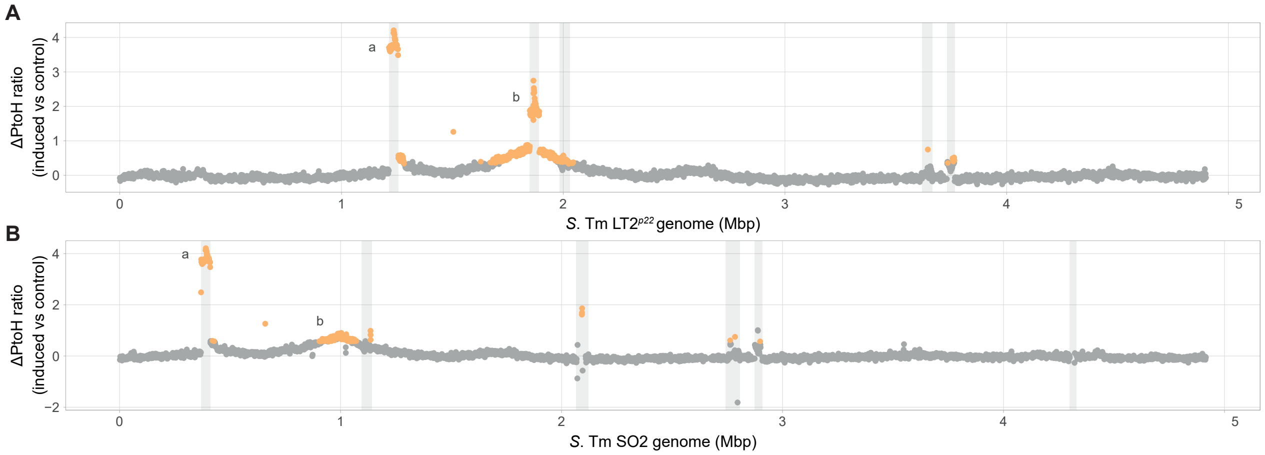

Supplementary Figure S1: A correct reference genome sequence is favourable for determining accurate ΔPtoH ratios.

ΔPtoH ratios shown as the mean log2-fold change (l2fc) of each gene in mitomycin C-induced versus control *S*.Tm LT2^{p22} cultures when aligning sequencing reads to the *S*. Tm LT2^{p22} genome (A) or the publicly available S. Tm SO2 (CP014356.1) genome (B). The data show that a reference genome from a close relative can be used to quantify prophage induction if the prophage is present as shown for p22 (a). However, the presence and inducibility of Fels-1 (b) would have been missed, and the effect of escape replication at the attL and attR sites of Fels-1 would have been underestimated. The areas highlighted in grey represent prophage regions predicted by PHASTER (left to right: (A) p22, Fels-1, Gifsy-2, Gifsy-1, Fels-2; (B) p22, Gifsy-2, Salmon_ST64B, Gifsy-1, Salmon_RE_2010, Salmon_SP_004). Statistical significance of l2fcs between induced and control cultures was tested as described for the main figures and in the Methods.

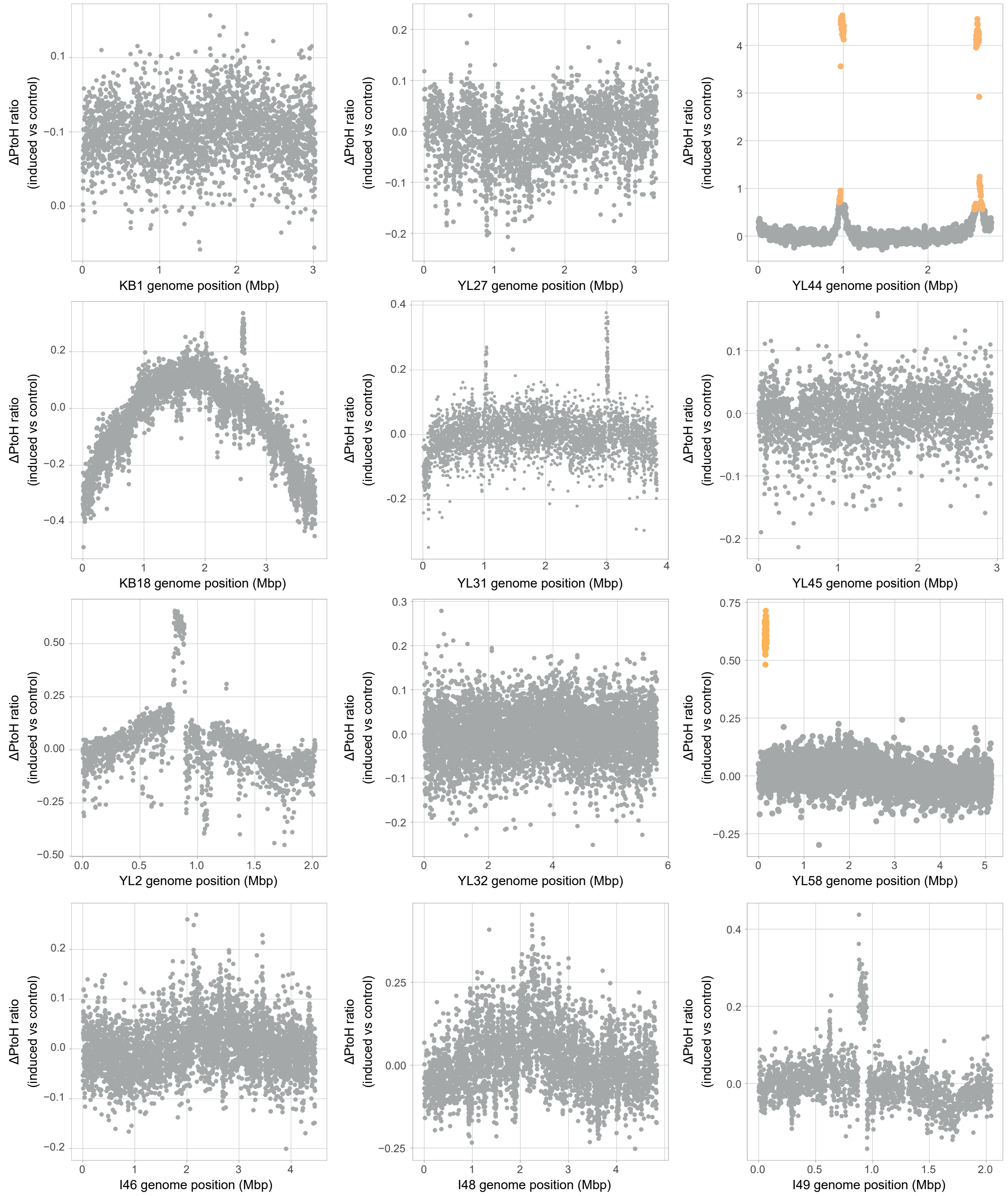

Supplementary Figure S2: Several *in vitro*-induced Oligo-MM^{12} strains reveal genomic regions with putative prophages.

ΔPtoH ratios of genes in mitomycin C-induced (n=3) and control (n=3) Oligo-MM^{12} strain cultures reveal genomic regions with putative prophages. In addition to *A. muciniphila* YL44 and *B. coccoides* YL58 that show significantly increased l2fc (orange dots) in prophage regions, several other strains show regions with an increased l2fc. Despite being statistically non-significant, likely due to variable degrees of induction across the three mitomycin C- induced replicates, those regions may still be indicative for additional inducible prophages or other mobile genetic elements. However, these regions were excluded from our analysis due to the stringent criteria we applied for identifying putative inducible phages. Statistical significance of l2fcs between induced and control cultures was tested as described for the main figures and in the Methods.

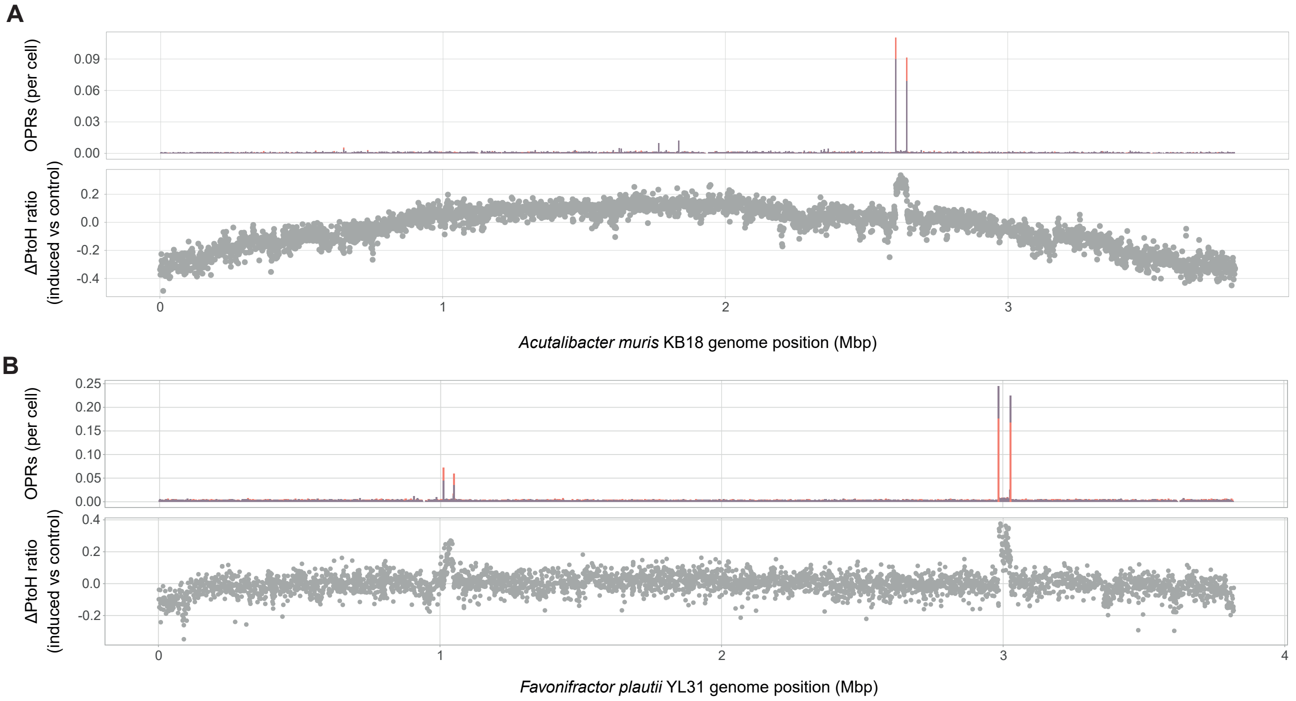

Supplementary Figure S3: OPR analysis locates a putative prophage region in cultured *A. muris* KB18 and *F. plautii* YL31.

Accumulation of OPRs after in vitro mitomycin C-induction revealed a putative prophage region in *A. muris* KB18 (A) and two region in *F. plautii* YL31 (B). No significant ΔPtoH ratio was observed for any of the putative prophage regions, which can be attributed to spontaneous induction as indicated by the presence of OPRs not only in induced (n=3) but also in control (n=3) cultures. For *F. plautii* YL31 phage 2 more OPRs were detected in the control than the induced cultures indicating that mitomycin C is not the primary inducer of this prophage.

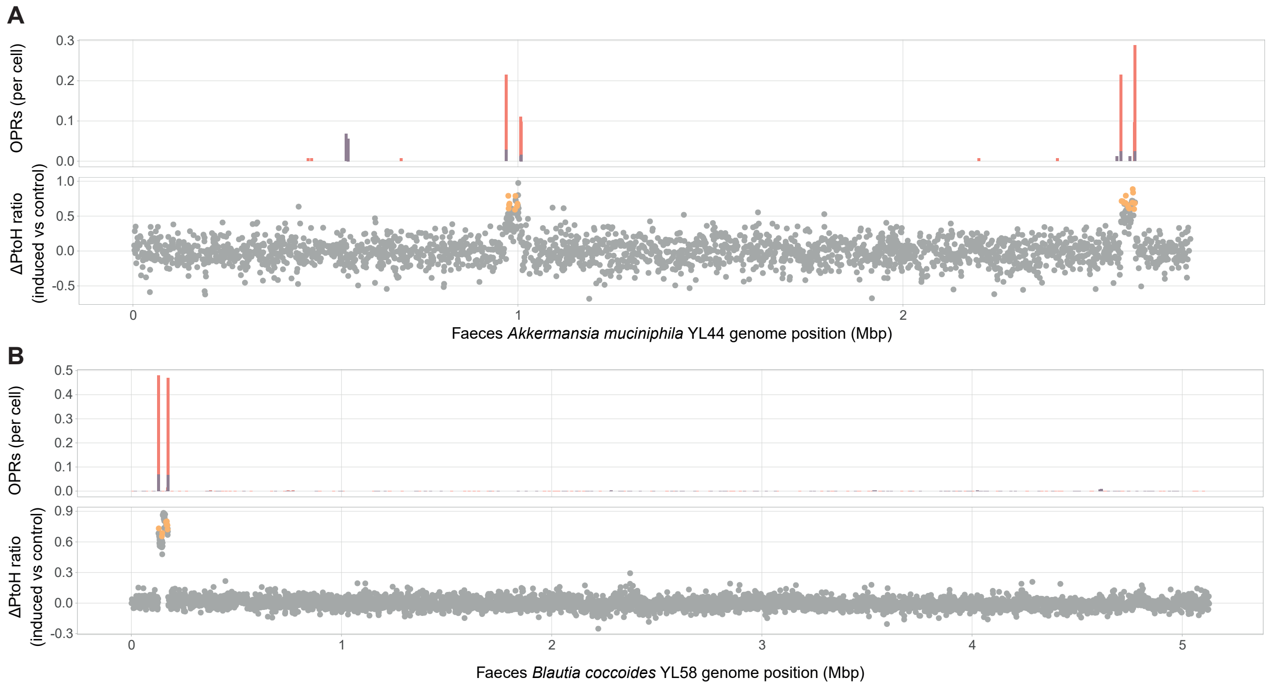

Supplementary Figure S4: *Ex vivo* induction of prophages in *A. muciniphila* YL44 and *B. coccoides* YL58. *Ex vivo* induced cultures of murine faecal pellets from Oligo-MM^{12} mice showed an accumulation of OPRs at the respective *att* sites of YL44 phage 1 and 2 (A: upper panel) and YL58 phage 1 (B: upper panel). Only a subset of genes from the three prophages (lower panel A and B) reveal a significant ΔPtoH ratio between mitomycin C-induced (n=3) and control (n=3) cultures, most likely due to inconsistent inductions across the experimental replicates. However, increased OPR counts at prophage borders indicate the location of induced prophages.
